# Investigations on the Genus *Rhizoecus* (Hemiptera: Rhizoecidae) with Description of Two New Species from South America

**DOI:** 10.1007/s13744-019-00681-w

**Published:** 2019-04-25

**Authors:** M B Kaydan, Z Konczné Benedicty, T Kondo, A A Ramos-Portilla, É Szita

**Affiliations:** 1 Plant Protection Institute, Centre for Agricultural Research, Hungarian Academy of Sciences, H-1022, Herman Ottó út 15, Budapest, Hungary; 2 Imamoglu Vocational School, Çukurova University, Adana, Turkey; 3 Biotechnology Research Centre, Çukurova University, Adana, Turkey; 4 Corporación Colombiana de Investigación Agropecuaria (AGROSAVIA), Centro de Investigación Palmira, Palmira, Valle Colombia; 5 Instituto Colombiano Agropecuario ICA, Grupo Sistemática de Insectos Agronomía, Museo Entomológico UNAB, Facultad de Agronomía, Universidad Nacional de Colombia, Bogotá, Colombia

**Keywords:** Hypogeic mealybugs, identification key, *Rhizoecus*, South America, taxonomy

## Abstract

Neotropical species of the scale insect genus *Rhizoecus* Künckel d’Herculais (Hemiptera: Coccomorpha: Rhizoecidae) found in soil sample material of the Hungarian Natural History Museum were studied. Two new *Rhizoecus* species, *Rhizoecus kontschani* Kaydan and Konczné Benedicty sp. n., and *Rhizoecus granaradewillinkae* Kaydan and Szita sp. n., are described and illustrated based on the adult females. Also, the adult females of *Rhizoecus keysensis* Hambleton and *Rhizoecus pseudocacticans* Hambleton are illustrated. An identification key and new additional locality records for the currently known *Rhizoecus* species are provided.

## Introduction

The rhizoecine mealybugs, formerly included in the Pseudococcidae, were elevated to family status, i.e., the Rhizoecidae (Hemiptera: Coccomorpha) by Hodgson (2012). The Rhizoecidae are mealybugs that mostly live underground and feed on plant rootlets and are commonly known as “root mealybugs” or “ground mealybugs” (Williams 1998). The family includes 238 extant species belonging to 19 genera (García Morales *et al*^2016^, Tanaka 2016) of which 69 species have been recorded from the Neotropical Region (Williams & Granara de Willink 1992, Kozár & Konczné Benedicty 2007, García Morales *et al*^2016^, Ramos-Portilla & Caballero 2016). The associated host plants of the rhizoecid mealybug species are often not clearly understood, and when they are known from different host plants, it is unclear whether they have any preference for any plants. According to the collecting method, specimens are often found loosely in the soil, not feeding on any host, and sometimes the same species may be found on the roots of many species of plants (A.A. Ramos-Portilla, personal observation). When sampling for rhizoecid mealybugs, it is often necessary to pull out the entire root system of the plant in order to determine whether the root on which the specimen was found belongs to the true host. As for species of the genus *Rhizoecus*, some degree of specialization can be found, where several species prefer xerophilous habitats (Kozár & Konczné Benedicty 2007).

Within the family Rhizoecidae, the subfamilies Xenococcinae and Rhizoecinae can be easily separated by the absence of ostioles, disc pores, and tubular ducts in the Xenococcinae, which are present in the Rhizoecinae. In the Rhizoecinae, three main lineages (tribes) can be recognized, namely the Geococcini, the Rhizoecini, and the Ripersiellini (Kozár & Konczné Benedicty 2007). The Geococcini is characterized by having (i) sclerotized anal lobes, (ii) strong setae on the head and posterior abdominal segments, and (iii) modified tritubular ducts. The Rhizoecini has tritubular ducts but without sclerotized anal lobes. Several new evolutionary lines are known in this tribe such as *Marottarhizoecus* Kozár & Konczné Benedicty which has tritubular ducts surrounded by multilocular pores, *Benedictycoccina* Kozár & Foldi which has tritubular ducts surrounded by trilocular pores, and *Coccidella* Hambleton which has a group of trilocular pore clusters in the middle of the ventral surface.

The Rhizoecini tribe is composed of 112 species grouped into six genera, namely *Benedictycoccina* Kozár and Foldi (4 spp.), *Coccidella* (10 spp.), *Kissrhizoecus* Kozár and Konczné Benedicty (1 sp.), *Rhizoecus* Künckel d’Herculais (93 spp.), *Marottarhizoecus* Konczné Benedicty (2 spp.), and *Williamsrhizoecus* Kozár & Konczné Benedicty (2 spp.) (Kozár & Konczné Benedicty 2007, García Morales *et al*^2016^, Kaydan *et al*^2018^).

The genus *Rhizoecus* has been studied in detail by different authors, some of which treated the genus in a broader sense (Ben-Dov 1994, Williams 1998, 2004, Jansen 2001, 2003) and others who considered the genus in a much narrower sense (Williams 1973, Matile-Ferrero 1976, Tang 1992, Kozár & Konczné Benedicty 2002, 2003, 2005, 2007, Kozár & Foldi 2004). According to Kozár and Konczné Benedicty (2007), *Rhizoecus* is characterized by the combination of the following features: (i) 5 or 6 segmented antennae, (ii) legs well developed, (iii) dorsum and venter with tritubular ducts, and (iv) anal ring with 6 setae.

*Rhizoecus* has 93 species of which 40 species occur in the Neotropical Region (Kozár & Konczné Benedicty 2007). In the present paper, two new *Rhizoecus* species are described from the Neotropical Region. An identification key and new additional locality records for the currently known *Rhizoecus* species present in the Neotropical Region are provided and discussed.

## Material and Methods

The specimens described and recorded in this study were all obtained from soil samples deposited in the Hungarian Natural History Museum (HNHM) collection (over 5000 samples in total). The samples were extracted by Berlese funnel, an apparatus widely used to extract living organisms, particularly arthropods, which works by creating a temperature gradient over the sample, such that mobile organisms will move away from the higher temperatures and fall into a collecting vessel, where they are preserved for examination (Southwood & Henderson 2000).

Specimens were prepared for light microscopy using the slide-mounting method discussed by Kosztarab & Kozár (1988). The morphological terminology used follows Williams (2004), Kozár and Konczné Benedicty (2007), and Ramos-Portilla (2014).

All measurements and counts were taken from all the available material and the values are given as a range for each character.

Type material is deposited in the Plant Protection Institute, Centre for Agricultural Research, Hungarian Academy of Sciences (PPI).

Distribution data for each species have been provided, with new country records signed with an asterisk. For the host plant list of each species, see García Morales *et al* (2016).

## Result and Discussion

### *Rhizoecus* Künckel d’Herculais

Type species: *Rhizoecus falcifer* Künckel d’Herculais, 1878, by monotypy

*Neorhizoecus* Hambleton, 1946

*Radicoccus* Hambleton, 1946

Body of adult female elongate to round, usually membranous. Anal lobe poorly or moderately developed, often bearing 3 long apical setae (1 ventral, 2 dorsal), or with numerous short setae. Antennae often placed close together, short, strongly geniculate, each 5 or 6 segmented, segments 2–5 often wider than long, last segment usually longer than wide; in species with 6-segmented antennae, the fifth segment has 1 falcate sensory seta and 4 falcate setae on the sixth antennal segment. In species with 5-segmented antennae, there are usually 5 falcate setae on the terminal segment and 1 falcate seta at the apex of the preapical segment. Labium longer than wide. Legs well developed, tarsus usually shorter than tibia, often with spine-like setae on inner margins, tarsus tapering, with a pair of knobbed digitules at apex. Claw slender and elongate, with short setose or knobbed digitules. Eyes present or absent. Circuli if present numbering 1 to 6, truncate, conical, elongate, cylindrical, or bulbous, with distal end sometimes flat, reticulated or with minute papilla-like projections. Anterior and posterior ostioles present, sometimes anterior ones absent.

Frons often with a ventral sclerotized cephalic plate, sometimes with a few setae on margin. Body setae usually short and hair-like, often extensively covering surface. Trilocular pores present. Oral collar tubular ducts and multilocular pores with 7–12 loculi present or absent. Anal ring well developed with or without long elongate triangular pores, bearing 6 hair-like setae. Internal female genital organ shows great variability in shape and size (Kozár & Konczné Benedicty 2007).

**Key to** ***Rhizoecus*** **species distributed in the Neotropics, based on adult females**

- 1 – Multilocular pores present.......................................2
– Multilocular pores absent......................................28
- 2 – Claw digitules spine-like, shorter than claw..................3
– Claw digitules with blunt apices, as long as claw..........................................*R. amorphophalli* (Betrem)
- 3 – Multilocular disc pores on dorsum present.............4
– Multilocular pores on dorsum absent....................20
- 4 – Oral collar tubular ducts on dorsum present............5
– Oral collar tubular ducts on dorsum absent...........10
- 5 – Oral collar tubular ducts in rows on all abdominal segments.....................................................................6
– Oral collar tubular ducts scarce on margin of dorsum only..............................*R. falcifer* (Kunckel d’Herculais)
- 6 – Antennae 6 segmented............................................7
– Antennae 5 segmented............................................8
- 7 – Multilocular disc pores present on the head and thorax on dorsum.............*R. iquitosi* Konczné Benedicty & Kozár
– Multilocular pores absent on the head and thorax on dorsum....................................*R. latus* (Hambleton)
- 8 – With a circulus....................................................................................*R. microtubularis* Konczné Benedicty & Kozár
– Without circulus.......................................................9
- 9 – Oral collar tubular ducts present on the head and thorax on venter........*R. boliviensis* Konczné Benedicty
– Oral collar tubular ducts absent on the head and thorax on venter.........................*R. nitidalis* Hambleton
- 10 – Anal ring outer pores with spiculae.........................11
– Anal ring outer pores without spiculae..................17
- 11 – Multilocular pores only on abdominal segments on dorsum.......................................................................12
– Multilocular pores on all over the surface on dorsum.......................................................................13
- 12 – Tritubular ducts short (about two times longer than wide).............................................*R. stangei* McKenzie
– Tritubular ducts long (about three-four times longer than wide)............................................*R. coffeae* Laing
- 13 – Anterior pair of ostioles absent.............................................................................*R. spinipes* (Hambleton)
– Anterior pair of ostioles present............................14
- 14 – One circulus present.............................................................*R. costaricensis* Konczné Benedicty & Kozár
– Circulus absent........................................................15
- 15 – Ostioles membranous, anal ring outer row pores with more than one spiculae.....................................16
– Ostioles sclerotized, anal ring outer row pores with one spiculus......................*R. americanus* (Hambleton)
- 16 – Tritubular ducts 46–54 altogether on both sides......................................*R. distinctus* (Hambleton)
– Tritubular ducts 60–74 altogether on both sides....................................*R. associatus* (Hambleton)
- 17 – Both pair of ostioles present..................................18
– Anterior pair of ostioles absent....................................................................................*R. granaradewillinkae* sp. n.
- 18 – Ostioles heavily sclerotized.................*R. caladii* Green
– Ostioles membranous...................................................19
- 19 – Multilocular disc pores mostly around vulva, very few on other parts of the venter and dorsum..............................*R. neomexicanus* McKenzie
– Multilocular disc pores in transverse rows and bands on both body surfaces.......................*R. kontschani* sp. n.
- 20 – Antennae 5 segmented..........................................21
– Antennae 6 segmented...............................................24
- 21 – Oral collar tubular ducts present on dorsum.........22
– Oral collar tubular ducts absent on dorsum..............23
- 22 – Circulus present..............................................................................................*R. erikae* Konczné Benedicty & Kozár
– Circulus absent.........................*R. pauciporus* Hambleton
- 23 – Trilocular pores present on dorsum.....................................*R. compotor* Williams & Granara de Willink
– Trilocular pores absent on dorsum........................................................................................*R. setosus* (Hambleton)
- 24 – Multilocular pores 4–10 around vulva...................25
– Multilocular pores 50–60 on at least last 3 abdominal segments.............................................................26
- 25 – Tritubular ducts short (about two times longer than wide).....................................*R. mayanus* (Hambleton)
– Tritubular ducts long (about three-four times longer than wide)....................*R. neostangei* Miller & McKenzie
- 26 – Oral collar tubular ducts present on last abdominal segment on venter, scarce........................................27
– Oral collar tubular ducts completely absent on venter.....................................*R. cyperalis* (Hambleton)
- 27 – Tritubular ducts present on venter...............................................................................*R. nemoralis* (Hambleton)
– Tritubular ducts absent on venter...........................................................................*R. subcyperalis* Hambleton
- 28 – Tubular ducts present...........................................29
– Tubular ducts absent..............*R. olmuensis* Hambleton
- 29 – With two or more circulus....................................30
– With one circulus...................................................32
- 30 – Three circuli present...............*R. ovatus* Hambleton
– Two circuli present.................................................31
- 31 – Tritubular ducts present on middle of thorax and head on dorsum, oral collar tubular ducts in transverse rows on abdominal segments..............*R. polyporus* Hambleton
– Tritubular ducts absent on middle of thorax and head on dorsum, oral collar tubular ducts very few on abdominal segments....................................................................*R. demerarae* Williams & Granara de Willink
- 32 – Tritubular ducts short (about two times longer than wide)..........................................................................33
– Tritubular ducts long (about three-four times longer than wide).................................................................38
- 33 – Anal ring pores with spiculae.................................34
– Anal ring without spiculae...........................................35
- 34 – Claw digitules spine-like, shorter than claw..........................................*R. arabicus* Hambleton
– Claw digitules with blunt apices, ca. same size as claw.........................................*R. keysensis* Hambleton
- 35 – Anal ring pores in outer row more than 30.............................................*R. macgregori* Hambleton
– Anal ring pores in outer row less than 26..............................................................................37
- 36 – Tritubular ducts present on middle part of last dorsal segments....................................*R. cacticans* (Hambleton)
– Tritubular ducts absent on middle part of last dorsal segments.............................*R. leucosomus* (Cockerell)
- 37 – Anal ring pores with spiculae......................................38
– Anal ring pores without spiculae................................40
- 38 – Number of tritubular ducts on dorsum between 30 and 40.................................*R. favacirculus* Hambleton
– Number of tritubular ducts on dorsum more than 41...............................................................................39
- 39 – Eyes present.........................*R. simplex* (Hambleton)
– Eyes absent...............................*R. tropicalis* Hambleton
- 40 – Claw digitules setose, longer than claw................41
– Claw digitules setose, shorter than claw...............................................................*R. variabilis* Hambleton
- 41 – Tritubular ducts on mid-dorsum present (more than 10).........................................*R. atlanticus* (Hambleton)
– Tritubular ducts on middorsum absent or very few (9 or fewer)......................................................................42
- 42 – Anal ring pores in outer row between 20 and 30............................................*R. chilensis* Hambleton
– Anal ring pores in outer row less than 20.............................................*R. apizacos* Hambleton

### *Rhizoecus arabicus* Hambleton

*Material examined*. Argentina: 4 females—Jujuy Province, Ledesma Department, Calilegua National Park, South of Abra de Cañas, moss forest, litter, 23°41.3′S, 64°54.1′W, 2253 m a.s.l., 05.11.2006, leg. Sziráki Gy, Horváth E, González Olazo E (HNHM D-Am 481; PPI: 12433)

*Distribution.* Bolivia, Colombia, Costa Rica, Guadeloupe, Mexico, Peru, Trinidad and Tobago, USA (García Morales *et al*^2016^); *Argentina

### *Rhizoecus boliviensis* Konczné Benedicty

*Material examined.* Bolivia: 2 females—Beni Department, Guayaramerin, Estancia Esperanza, banana plantation, dry, decaying roots below preceding layer, 07.12.1965, leg. Balogh J, Mahunka S, Zicsi A (HNHM D-Am 2866; PPI: 12790); 1 female—same place and collectors, cacao plantation, lower, rooty horizon of leaf litter, 10.10.1965 (HNHM D-Am 2868; PPI: 12461); 1 female—same place and collectors, untouched forest, humid leaf litter, 10.10.1965 (HNHM D-Am 2869; PPI: 12462); 3 females—same place and collectors, gallery forests along the Mamore river, lower horizon of leaf litter, 10.10.1965 (HNHM D-Am 2872; PPI: 12463)

*Distribution.* Bolivia (Kozár & Konczné Benedicty 2007)

### *Rhizoecus cacticans* (Hambleton)

*Material examined.* Bolivia: 1 female—Beni Department, Guayaramerin, Estancia Esperanza, untouched forest, humid leaf litter, 10.10.1965, leg. Andrásy I, Balogh J, Loksa I, Mahunka S, Zicsi A (HNHM D-Am 2869; PPI: 12462). Chile: 1 female—Provincia Santiago, Tiltil, Cuesta la Dormida, drier parts of valley side, soil, 05.11.1965, leg. Balogh J, Mahunka S, Zicsi A (HNHM D-Am 2861; PPI: 12791)

*Distribution.* Argentina, Australia, Chile, Colombia, Czech Republic, Denmark, Ecuador, France, Germany, Greece, Guatemala, Honduras, Hungary, Italy (mainland and Sicily), Netherlands, Norway, Peru, Poland, Russia, Spain (Canary Islands), UK, USA (García Morales *et al*^2016^); *Bolivia

### *Rhizoecus costaricensis* Konczné Benedicty & Kozár

*Material examined.* Brazil: 1 female—Maranhão State, northwest from Imperatriz, Serra do Gurupi, Fazenda Agua Azul, leaf litter, 09.09.1967, leg. Balogh J (HNHM D-Am 2916; PPI: 12793). Colombia: 2 females—Rio Carlo, leaf litter and moss, 02.10.1984, leg. Zicsi A, Loksa I (HNHM Colombia, 1984.X.; PPI: 12431). Costa Rica: 3 females—Heredia Province, La Selva Biological Station, River Sura, primary rain forest, 800 m a.s.l., 14.01.1992, leg. Balogh J (HNHM CR92.B21; PPI: 12792). Venezuela: 1 female—Maracay University, park, leaf litter and root matrix, 30. 08.1973 (HNHM D-Am 3311; PPI: 12794)

*Distribution.* Bolivia, Costa Rica (Kozár & Konczné Benedicty 2007), *Colombia, *Venezuela

### *Rhizoecus demerarae* Williams and Granara de Willink

*Material examined.* Argentina: 3 females—Jujuy Province, Ledesma Department, Calilegua National Park, South of Abra de Cañas, moss forest, litter and moss, 1700 m a.s.l., 05.11.2006, leg. Sziráki Gy, Horváth E, González Olazo E (HNHM D-Am 469; PPI: 12798); 1 female—same data (HNHM D-Am 470. PPI: 12799). Brazil: 2 females—São Paulo State, Campinas, Americana, leaf litter, 21.09.1967, leg. Balogh J (HNHM D-Am 2927; PPI: 12803), 1 female—Rio de Janeiro State, Itatiaia National Park, Itaporani rainfall, primary rain forest, leaf litter, soil and moss, 27.05.1992, leg. Balogh J (HNHM BR92.B.5; PPI: 12795); 2 females—Mato Grosso State, Pantanal, Fazenda Uberaba, Garon Maya, leaf litter, leg. Balogh J (HNHM BR92.B.55; PPI: 12797); 1 female—São Paolo State, São Roqūe, Project Itatūba, Eucalyptus plantation, leaf litter, 850 m a.s.l., 09.01.1995, leg. Balogh J (HNHM BR95.B17; PPI: 12796); 5 females—São Paolo State, São Roque, Project Itatuba, Sapucaia Lake, submontane rain forest, leaf litter, 800 m a.s.l., 09.01.1995, leg. Balogh J (HNHM BR95.B21; PPI: 12396). Colombia: 1 female—Rio Carlo, leaf litter and moss, 02.10.1984, leg. Zicsi A, Loksa I (HNHM Colombia, 1984; PPI: 12432). Ecuador: 1 female—Pichincha Province, above Quito, 46 km leaving Quito to Santo Domingo, soil from 15-cm depth, 3200–3400 m a.s.l., 21.04.1988, leg. Zicsi A, Csuzdi Cs (HNHM D-Am 634; PPI: 12800); 2 females—Pichincha Province, on the way from Tandajapa to Nono, rainforest, moss, 08.04.1987, leg. Zicsi A, Loksa I (HNHM D-Am 715; PPI: 12802); 1 female—Azuay Province, leaving Chordeleg, 39 km from Cuenca, moss, 03.05.1988, leg. Zicsi A, Csuzdi Cs (HNHM D-Am 676; PPI: 12801); 4 females—Azuay Province, 2 km leaving Sigsig, moss from the slope of bank, 03.05.1988, leg. Zicsi A, Csuzdi Cs (HNHM D-Am 687; PPI: 12448)

*Distribution.* Brazil, Guayana, Peru (García Morales *et al*^2016^); *Argentina, *Colombia, *Ecuador

### *Rhizoecus distinctus* (Hambleton)

*Material examined.* Costa Rica: 2 females—Heredia Province, La Selva Biological Station, River Sura, primary rain forest, 800 m a.s.l., 14.01.1992, leg. Balogh J (HNHM Cr92.B.21; PPI: 12804); 1 female—Talamanca Mt. Range, Sierra de La Muerte, El Empalme, lower mountain wet forest, leaf litter and root matrix, 2150 m a.s.l., 24.01.1992, leg. Balogh J (HNHM Cr92.B.61; PPI: 12805)

*Distribution.* USA (García Morales *et al*^2016^); *Costa Rica

### *Rhizoecus falcifer* Künckel d’ Herculais

*Material examined.* Chile: 1 female—Tarapaca Province, Misituni, Berlese-samples from 12 points in cross-section of smaller valley running at right angles to Rio Lauca, 25.11.1965, leg. Andrásy I, Balogh J, Loksa I, Mahunka S, Zicsi A (HNHM D-Am 2716; PPI: 12809). Costa Rica: 2 females—Alajuela Province, Arenal volcano, hot springs, secondary rain forest leaf litter, 2800 m a.s.l., 16.01.1993, leg. Balogh J (HNHM CR93.B112; PPI: 12806); 1 female—Puntarenas Province, Manuel Antonio National Park, secondary rain forest, leaf litter and root matrix, 24.01.1993, leg. Balogh J (HNHM CR93.B133; PPI: 12807); 1 female—same data (HNHM CR93 B136; PPI: 12419); 5 females—same data (HNHM CR93 B141; PPI: 12421). Ecuador: 1 female—Prov. Azuay, 26 km from Cuenca, grass, 26.04.1988, leg. Zicsi A, Csuzdi Cs (HNHM D-Am 599; PPI: 12808)

*Distribution.* Algeria, Australia, Czech Republic, France, Hungary, Ireland, Italy (mainland and Sicily), Malta, Mexico, Morocco, New Zealand, Saint Helena, South Africa, Spain (mainland and Canary Islands), Suriname, Trinidad and Tobago, Trinidad, UK, England, USA (García Morales *et al*^2016^); *Chile, *Costa Rica, *Ecuador

### *Rhizoecus granaradewillinkae* Kaydan & Szita sp. n. (Fig 1)

*Material examined.* Costa Rica: Holotype: 1 female—Alajuela Province, Arenal volcano, Northern slopes, rainforest, moss from trees, 400–500 m a.s.l., 16.01.1993, leg. Balogh J (HNHM CR93.B108; PPI: 12837)

**Fig 1:**
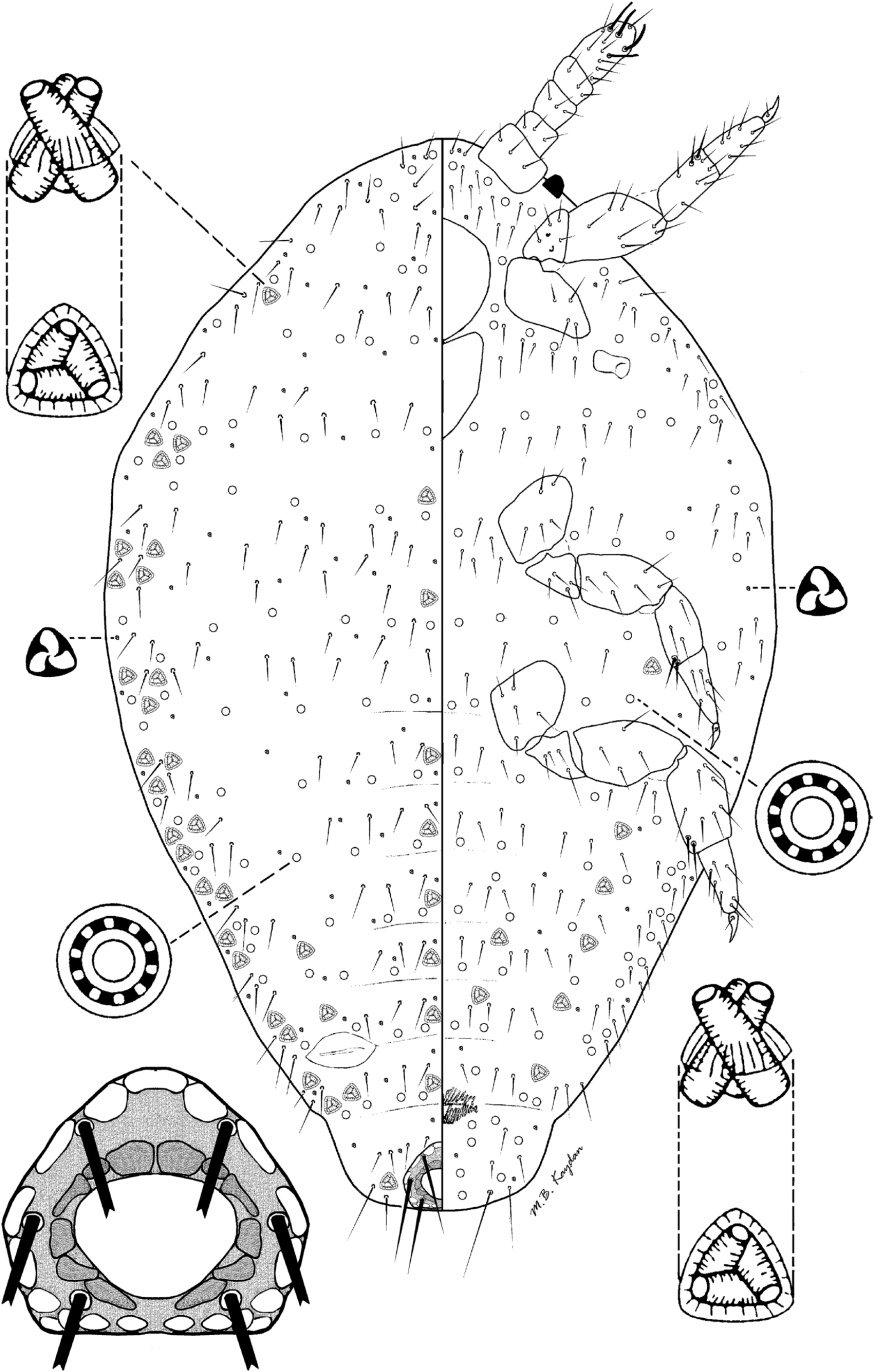
*Rhizoecus granaradewillinkae* Kaydan and Szita sp. n., adult female.

*Distribution.* Costa Rica

*Description.* Slide-mounted adult female

*Adult female.* Body elongate-oval, 0.6 mm long, 0.3 mm wide. Eyes marginal, 8–10 μm wide. Antenna 5 segmented, 115–120 μm long, with 4 fleshy setae, on apical segment each seta 25–28 μm long; apical segment 40–43 μm long, 22.5 μm wide, with apical setae each 25–28 μm long. Clypeolabral shield 75 μm long, 70 μm wide. Labium 3 segmented, 50 μm long, 45 μm wide. Anterior spiracles each 15–18 μm long, 7–10 μm wide across atrium; posterior spiracles each 20–25 μm long, 7–10 μm wide across atrium. Legs well developed, length data for posterior legs: coxa 45–60 μm, trochanter + femur 90 μm, tibia + tarsus 102–105 μm, claw 15–18 μm. Ratio of lengths of tibia + tarsus to trochanter + femur 1.13–1.16:1; ratio of lengths of tibia to tarsus 1.00–1.40:1; ratio of length of hind trochanter + femur to greatest width of femur 2.00–2.04:1. Claw digitules spine-like, 5 μm long. Anterior ostioles not detected; posterior ostioles without trilocular pores and with only 3 setae. Anal ring 40.0 μm wide, bearing 6 setae, each seta 75–95 μm long

*Dorsum*. Derm membranous, without any cerarii around body margin. Setae on anal lobe hair-like, each 40–45 μm long; body setae short, flagellate, each 10–25 μm long, scattered on the head, thorax, and abdominal segments. Trilocular pores each 2.0–2.5 μm in diameter, scattered over the entire body. Multilocular disc pores on abdominal segments as follows: segments I–III, 27; IV, 9; V, 8; VI, 8; VII, 9; VIII + IX, 0; and about 74 scattered on the head and thorax; each pore 9–10 μm in diameter. Tritubular ducts, each duct 7–8 μm wide at mid-width, on abdominal segments as follows: segment I: 5, II: 6, III: 5, IV: 5, V: 6, VI: 5, VII: 5, VIII + IX: 2; and 30 on the thorax and head; each pore 8–9 μm in diameter

*Venter*. Setae flagellate, each 10–25 μm long, longest setae situated medially on the head. Apical setae of anal lobe each 55–60 μm long. Multilocular disc pores on abdominal segments as follows: segments I–III, 30; IV, 9; V, 9; VI, 7; VII, 12; VIII + IX, 6; and scattered on the head and thorax: 84; each pore 7–8 μm in diameter. Trilocular pores, each 2.5 μm in diameter, scattered on venter. Tritubular ducts, each duct 5 μm wide at mid-width, present in a single row across abdominal segments, as follows: segments I–III, 2; IV, 6; V, 5; VI, 5; VII, 5; VIII + IX, 2

*Etymology.* The species was named in honor of the Argentinean coccidologist, María Cristina Granara de Willink, who made significant contributions to the knowledge of the taxonomy and fauna of South American scale insect species.

*Comments. Rhizoecus granaradewillinkae* Kaydan & Szita is characterized by having (i) five segmented antennae, (ii) claw digitules spine-like, (iii) only posterior pairs of ostioles present, (iv) multilocular pores present on both venter and dorsum, and (v) absence of oral collar tubular ducts on both sides. *Rhizoecus granaradewillinkae* is most similar to *R. distinctus* (Hambleton) and *R. associatus* (Hambleton) in having multilocular pores on dorsum and spine-like claw digitules shorter than claw, while the new species *Rhizoecus granaradewillinkae* differs from all the above species in lacking anterior ostioles.

### *Rhizoecus keysensis* Hambleton (Fig 2)

*Material examined.* Chile: 2 females—Provincia Valparaiso, Concón, 5 km from Concón on the road leading to Quintero, sand dunes, beneath, tean-tree, 10.10.1965, leg. Andrásy I, Balogh J, Loksa I, Mahunka S, Zicsi A (HNHM D-Am 2726; PPI: 12457). Ecuador: 2 females—Pichincha Province, Pululagua crater and its surroundings, Mitad del Mundo, moss from under bushes growing on the sides of the hollow, 12.02.1986, leg. Zicsi A, Loksa I (HNHM D-Am 592; PPI: 12434); 1 female—Azuay Province, between Giron and Victoria del Portete, leaf litter, 02.05.1988, leg. Zicsi A, Csuzdi Cs (HNHM D-Am 686; PPI: 12810)

**Fig 2:**
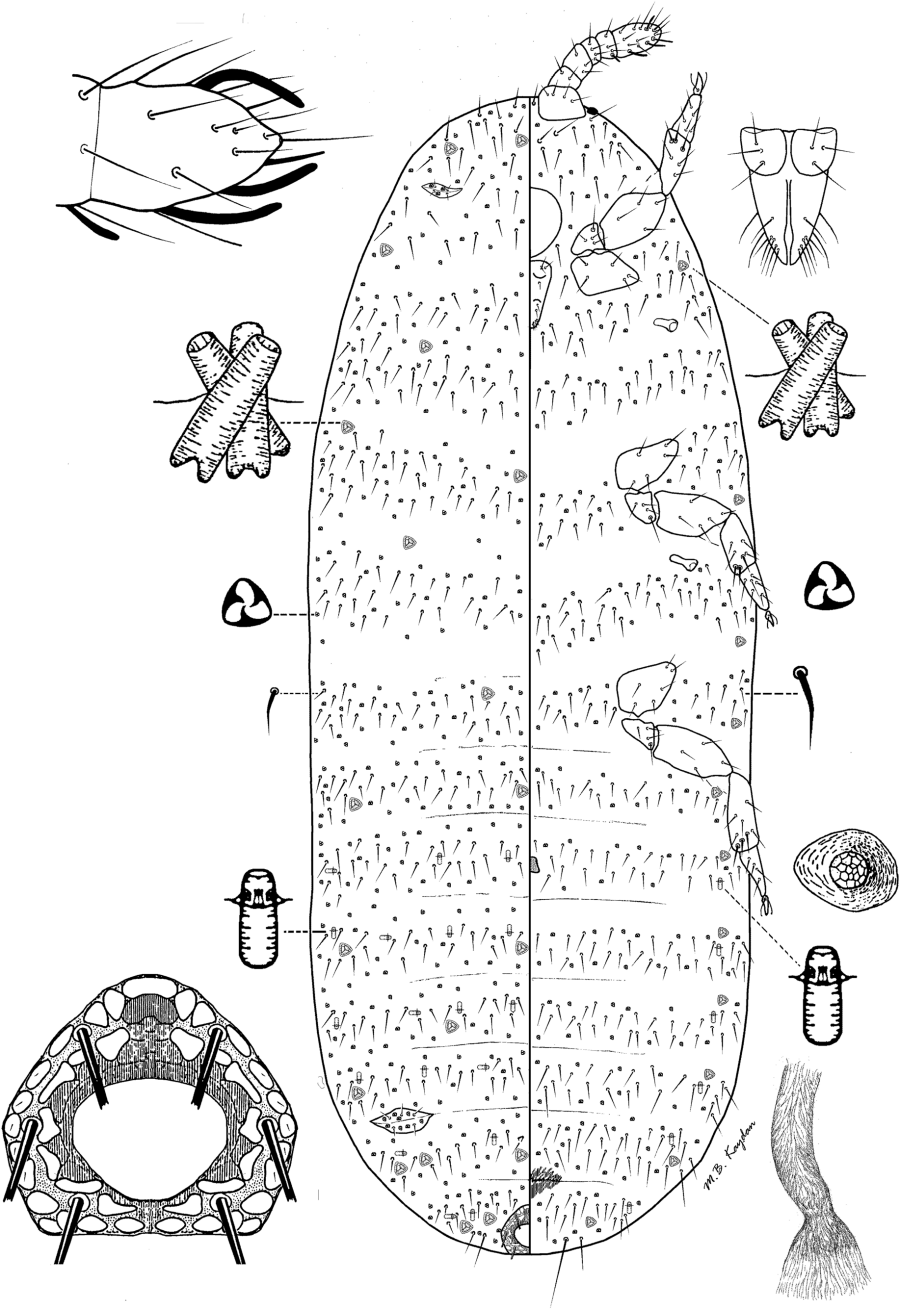
*Rhizoecus keysensis* Hambleton, adult female.

*Distribution.* USA (García Morales *et al*^2016^), *Chile, *Ecuador

### *Rhizoecus kontschani* Kaydan & Konczné Benedicty sp. n. (Fig 3)

*Material examined.* Holotype: Brazil—1 female—Rio de Janeiro State, Itatiaia National Park, Itaporani rainfall, moss, 27.05.1992, leg. Balogh J (HNHM BR92.B9; PPI: 12360). Paratypes: 4 females in 3 slides (1,1,2)—same data as holotype; 8 females in 3 slides (3,3,2)—São Paolo State, Ilha do São Sebastiaõ, protected urban forest, leaf litter, 29.05.1992, leg. Balogh J (HNHM BR92.B14; PPI: 12366); 1 female—São Paolo State, Caraguatatuba, Serra do Mar State Park, Atlantic rain forest, moss, 900–1000 m a.s.l., 03.06.1992, leg. Balogh J (HNHM Br92.B39; PPI: 12831)

**Fig 3:**
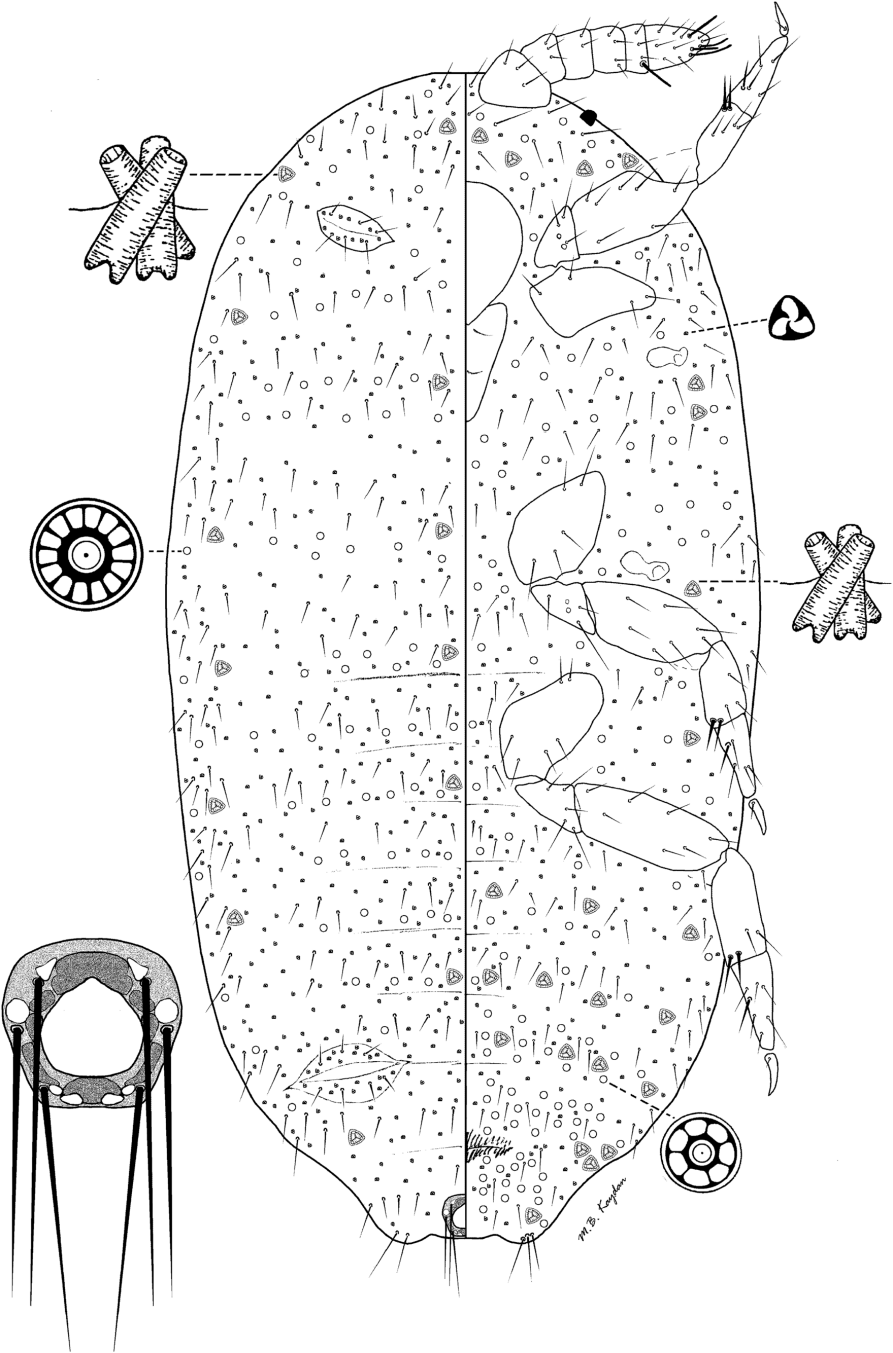
*Rhizoecus kontschani* Kaydan and Konczné Benedicty sp. n., adult female.

*Distribution.* Brazil

*Description.* Slide-mounted adult female

*Adult female.* Body elongate-oval, 0.80–1.56 mm long, 0.42–0.94 mm wide. Eyes marginal, 7.5–10.0 μm wide. Antenna 6 segmented, 150–170 μm long; apical segment 35–45 μm long, 30–35 μm wide, with 4 fleshy setae (plus 1 fleshy seta on fifth segment), each seta 30–40 μm long and apical setae each 25.0–27.5 μm long. Clypeolabral shield 85–100 μm long, 85.0–87.5 μm wide. Labium 3 segmented, 95–100 μm long, 60–65 μm wide. Anterior spiracles each 32.0–35 μm long, 12–15 μm wide across atrium; posterior spiracles each 35–38 μm long, 15–20 μm wide across atrium. Legs well developed, length data for posterior legs: coxa 85–100 μm, trochanter + femur 140–160 μm, tibia + tarsus 130–165 μm, claw 30–35 μm. Ratio of lengths of tibia + tarsus to trochanter + femur 1.01–1.00:1; ratio of lengths of tibia to tarsus 0.96–1.0:1; ratio of length of hind trochanter + femur to greatest width of femur 2.30–3.1:1. Claw digitules spine-like, 3 μm long. Both pairs of ostioles present; anterior ostioles each with a total for both lips of 10–16 trilocular pores and 6–10 setae; posterior ostioles each with a total for both lips of 12–18 trilocular pores and 7–10 setae. Anal ring 45–55 μm wide, bearing 6 setae, each seta 65–90 μm long

*Dorsum*. Derm membranous, without any cerarii around body margin. Setae on anal lobe hair-like, each 55–60 μm long; body setae short, flagellate, each 10–25 μm long, scattered on the head, thorax, and abdominal segments. Trilocular pores each 2–3 μm in diameter, scattered over the entire body. Multilocular disc pores on abdominal segments numbering as follows: segment I, 8–11; II, 10 or 11; III, 9–13; IV, 7–9; V, 5–9; VI, 0–2; VII, 0–2; VIII + IX, 0–4; 72–97 pores scattered on the thorax and head, each pore 6–8 μm in diameter. Tritubular ducts present over the entire body and form a submarginal and a median row, having 3 or 4 ducts on each abdominal segments, except on segment VIII + IX, each duct 10–12 μm wide on medial row

*Venter*. Setae flagellate, each 10–35 μm long, longest setae situated medially on the head. Apical setae of anal lobe each 55–60 μm long. Multilocular disc pores on abdominal segments numbering as follows: segments I–III, 42–44; IV, 13–15; V, 18–28; VI, 23–30; VII, 41–48; VIII + IX, 44–46; 81–102 pores scattered on the thorax and head, each pore 7–8 μm in diameter. Trilocular pores, scattered, each 2.0–2.5 μm in diameter. Tritubular ducts, each 7.5–8.0 μm wide at mid-width, present in a single row across abdominal segments, as follows: segment IV, 5; V, 7; VI, 8; on margin of segment VII, 4–6; VIII + IX, 2; I–III, 10–12; and head and thorax 10–14

*Etymology.* The species was named after the Hungarian acarologist, Jenő Kontschán, who gave great motivation to work on South American materials.

*Comments. Rhizoecus kontschani* Kaydan & Konczné Benedicty is characterized by having (i) six segmented antennae, (ii) claw digitules spine-like, (iii) anterior and posterior pairs of ostioles present, (iv) multilocular pores present on both venter and dorsum, and (v) absence of oral collar tubular ducts on both sides. *Rhizoecus kontschani* Kaydan & Konczné Benedicty is closely related to *R. spinipes* (Hambleton), *R. distinctus* (Hambleton), and *R. associatus* (Hambleton), in having multilocular pores on dorsum, spine-like claw digitules shorter than claw and lacking tubular ducts. But the new species *Rhizoecus kontschani* Kaydan & Konczné Benedicty differs from all the above species in having high number or multilocular disc pores scattered on dorsum.

### *Rhizoecus macgregori* Hambleton

*Material examined.* Bolivia: 2 females—La Paz Department, Caranavi Province, between Corocio and La Paz, after Umduari, shrubby vegetation, leaf litter and moss, 3200 m a.s.l., 16.11.1971, leg. Balogh J (HNHM D-Am 2986; PPI: 12821); 1 female—La Paz Department, Caranavi Province, between Puerto Linares and Caranavi, 41 km from Puerto Lineares, mountain forest, leaf litter, 14.11.1971, leg. Balogh J (HNHM D-Am 2993; PPI: 12822); 1 female—same data (HMHM D-Am 2994. PPI: 12823); 1 female—same data (HNHM D-Am 2995; PPI: 12824). Chile: 1 female—Santiago Province, Maipu, Quebrada, La Plata, Pundo: La Rinconada, 25 km SW from Santiago de Chile, leaf litter from thick, dry forest along brook, 28.09.1965 (HNHM D-Am 2755; PPI: 12819); 2 females—Valparaiso Province, Concón, 5 km from Concón on the road leading to Quintero, bank of lake among sand dunes, leaf litter of lake-side trees, 10.10.1965, leg. Andrásy I, Balogh J, Loksa I, Mahunka S, Zicsi A (HNHM D-Am 2726; PPI: 12458); 1 female—same data (HNHM D-Am 2735; PPI: 12818); 1 female—Coquimho Provincia, Los Villos, Berlese-samples from jungle, leaf litter and soil from drier spot, 05.12.1965, leg. Andrásy I, Balogh J, Loksa I, Mahunka S, Zicsi A (HNHM D-Am 2836; PPI: 12820)

*Distribution.* USA (Hambleton 1976), *Chile, *Ecuador

### *Rhizoecus microtubularis* Konczné Benedicty & Kozár

*Material examined.* Costa Rica: 1 female—Alajuela Province, Poas Volcano National Park, leaf litter and soil, 1800 m a.s.l., 21.01.1992., leg. Balogh J (HNHM CR 92.B 51; PPI: 12404); 1 female—Talamanca Mt. Range, Sierra de La Muerte, Alto de la Gloria, lower mountain wet forest, leaf litter, 1800 m a.s.l., 24.01.1992, leg. Balogh J (HNHM CR 92.B54; PPI: 12811); 1 female—same data, dry leaf litter of epiphytons (HNHM CR 92.B60; PPI: 12812)

*Distribution.* Costa Rica, Mexico (Kozár & Konczné Benedicty 2007)

### *Rhizoecus neostangei* Miller & McKenzie

*Material examined.* Brazil: 3 females—Serra do Mar Mt. Range, Rio de Janeiro State, near Paraty, Atlantic forest, leaf litter, 300 m a.s.l., 05.12.1990, leg. Balogh J (HNHM Br90.B.95; PPI: 12813)

*Distribution.* Mexico (García Morales *et al*^2016^), *Brazil

### *Rhizoecus nitidalis* Hambleton

*Material examined.* Brazil: 1 female—Serra do Mar Mt. Range, Rio de Janeiro State, near Paraty, Atlantic forest, leaf litter, 300 m a.s.l., 05.12.1990, leg. Balogh J (HNHM Br90.B94; PPI: 12814)

*Distribution.* Brazil (García Morales *et al*^2016^)

### *Rhizoecus ovatus* Hambleton

*Material examined.* Bolívia: 1 female—La Paz Department, Caranavi Province, between Corocio and La Paz, moss forest, litter, 2800 m a.s.l., 16.11.1971, leg. Balogh J (HNHM D-Am 2971; PPI: 12815)

*Distribution.* Mexico (García Morales *et al*^2016^), *Bolivia

### *Rhizoecus polyporus* Hambleton

*Material examined.* Bolivia: 2 females—Beni Dept., 10 km W of Guayaramerin, along the road to Riberalta, virgin forest, sandy soil, leaf litter and root matrix, 28.11.1966, leg. Balogh J (HNHM D-Am 2878; PPI: 12817). Brazil: 1 female—Sao Paolo State, Sao Roqūe, Project Itatūba, *Eucalyptus* plantation, leaf litter, 09.01.1995, leg. Balogh J (HNHM BR95.B19; PPI: 12816)

*Distribution.* Mexico (García Morales *et al*^2016^), *Bolivia, *Brazil

### *Rhizoecus pseudocacticans* Hambleton (Fig 4)

*Material examined.* Bolivia: 2 females—La Paz Department, Caranavi Province, between Corocio and La Paz, after Umduari, shrubby vegetation, leaf litter and moss, 3200 m a.s.l., 16.11.1971, leg. Balogh J (HNHM D-Am 2986; PPI: 12821); Chile: 3 females—Valparaiso Province, Concón, 5 km from Concón on the road leading to Quintero, bank of lake among sand dunes, leaf litter of lake-side trees, 10.10.1965, leg. Andrásy I, Balogh J, Loksa I, Mahunka S, Zicsi A (HNHM D-Am 2726; PPI: 12458); 1 female—same data (HNHM D-Am 2735; PPI: 12818); 2 females—Coquimho Provincia, Los Villos, Berlese-samples from jungle, leaf litter and soil from drier spot, 05.12.1965, leg. Andrásy I, Balogh J, Loksa I, Mahunka S, Zicsi A (HNHM D-Am 2836; PPI: 12820)

**Fig 4:**
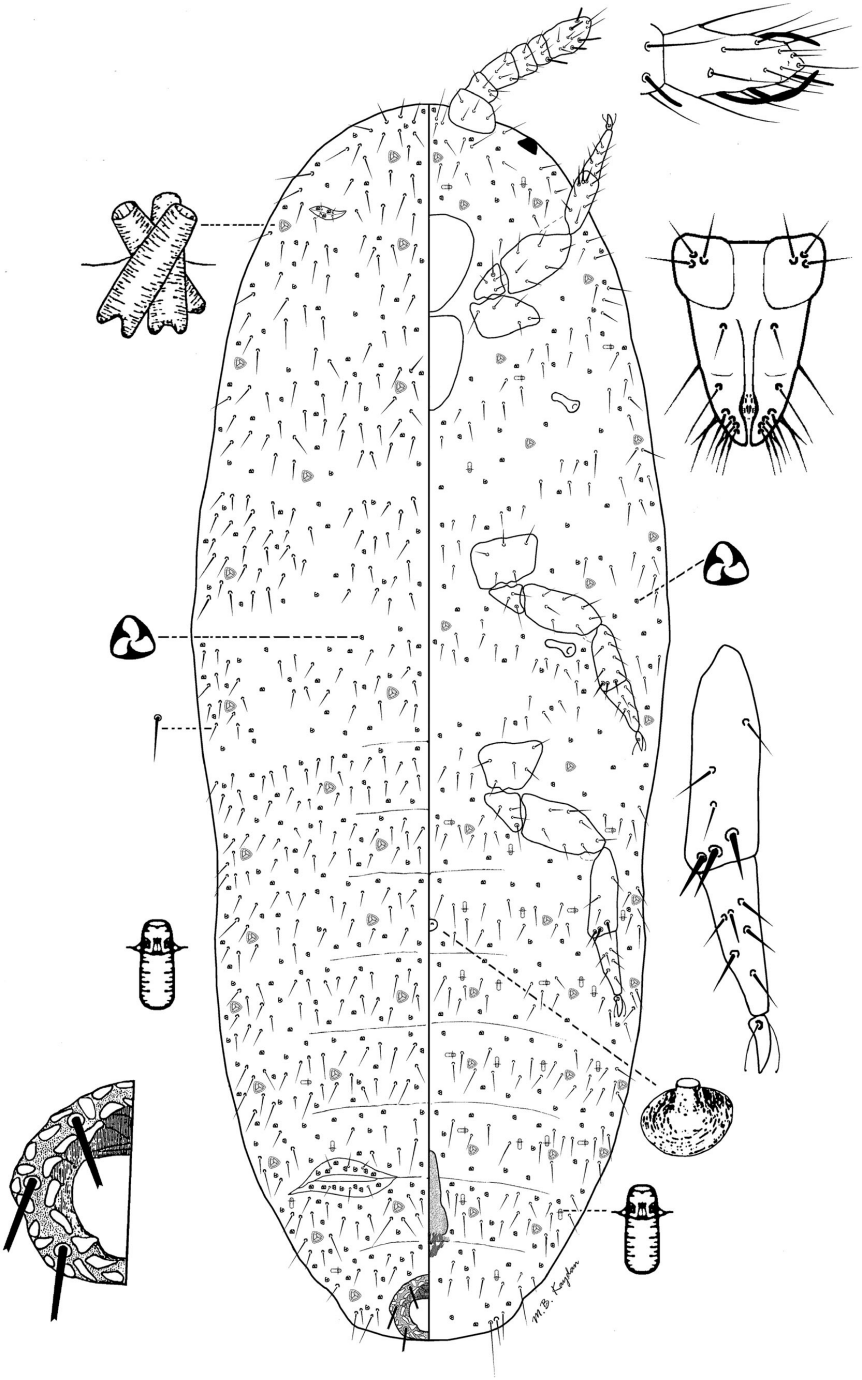
*Rhizoecus pseudocacticans* Hambleton, adult female.

*Distribution.* USA (García Morales *et al*^2016^), no. 4298}, Spain (Canary Islands) (Kaydan *et al*^2016^), *Bolivia, *Chile

### *Rhizoecus setosus* (Hambleton)

*Material examined.* Bolivia: 1 female—La Paz Department, Caranavi Province, between Puerto Linares and Caranavi, 41 km from Puerto Lineares, mountain forest, leaf litter, 14.11.1971, leg. Balogh J (HNHM D-Am 2994; PPI: 12826). Ecuador: 2 females—Napo Province, Rio Jondachi, somewhat after leaving Jondachi, riverside forest, soil and leaf litter, 10.04.1987., leg. Zicsi A, Loksa I (HNHM D-Am 709PPI: 12825); 2 females—same data (HNHM D-Am 729; PPI: 12456). Peru: 2 females—Lima-Pucallpa transect, 212 km from Pucallpa, mountain rain forest, moss and leaf litter, 02–04.11.1971, leg. Balogh J (HNHM D-Am 3030; PPI: 12827); 1 female—same data (HNHM D-Am 3031; PPI: 12828)

*Distribution****.*** Colombia, Ecuador, Peru (García Morales *et al*^2016^), *Bolivia

### *Rhizoecus spinipes* Hambleton

*Material examined.* Costa Rica: 1 female—Talamanca Mt. Range, Sierra de La Muerte, Alto de la Gloria, dry leaf litter of epiphytons, 1800 m a.s.l., 24.01.1992, leg. Balogh J (HNHM CR92.B60; PPI: 12835); 4 females—Cartago Province, Turrialba, Tropical Agronomy Research and Learning Center (CATIE), moss from trunks, 150 m a.s.l.,12.01.1993, leg. Balogh J (HNHM CR93.B74. PPI: 12833); 1 female—Limón Province, near Guápiles, Atlantic rain forest, leaf litter and root matrix, 400–500 m a.s.l., 12.01.1993, leg. Balogh J (HNHM CR93.B82; PPI: 12834); 1 female—Puntarenas Province, Manuel Antonio National Park, secondary rain forest, leaf litter and root matrix, 24.01.1993, leg. Balogh J (HNHM CR93.B141; PPI: 12424)

*Distribution.* Mexico, USA (García Morales *et al*^2016^), *Costa Rica

### *Rhizoecus subcyperalis* Hambleton

*Material examined.* Costa Rica: 1 female—San José Province, Cerro La Muerte, mountain rain forest, moss and leaf litter, 3400 m a.s.l., 24.01.1992, leg. Balogh J (HNHM CR92.B69; PPI: 12838)

*Distribution.* USA (García Morales *et al*^2016^), *Costa Rica

### *Rhizoecus variabilis* Hambleton

*Material examined.* Brazil: 1 female—São Paolo State, Sao Roqūe, Project Itatūba, *Eucalyptus* plantation, leaf litter, 09.01.1995, leg. Balogh J (HNHM Br95.B19; PPI: 12839); 3 females—Serra del Caldas Navas, Serrado, Caldas Navas, soil and root matrix, 24.01.1995, leg. Balogh J (HNHM Br95.B55; PPI: 12840); 1 female—Rio de Janeiro State, Itatiaia National Park, Itaporani rainfall, rain forest, leaf litter, 1400 m a.s.l.,16.02.1995, leg. Balogh J (HNHM BR95.B83; PPI code: 12836). Chile: 2 females—Tarapaca Province, Misituni, Berlese-samples from 12 points in cross-section of smaller valley running at right angles to Rio Lauca,,25.11.1965, leg. Andrásy I, Balogh J, Loksa I, Mahunka S, Zicsi A (HNHM D-Am 2716; PPI code: 12776); 1 female—Santiago Province, El Arrayan, 10 km E from Santiago de Chile, fern stems and moss from the ground, 9.10.1965, leg. Andrásy I, Balogh J, Loksa I, Mahunka S, Zicsi A (HNHM D-Am 2724; PPI: 12843); Ecuador: 2 females—Loja Province, leaving Saraguro, 175 km from Cuenca, moss, 26.04.1988, leg. Zicsi A & Csuzdi Cs (HNHM D-Am 612; PPI: 12841); 1 female—same data, soil (HNHM D-Am 621; PPI: 12842)

*Distribution.* Colombia, Guadalupe (García Morales *et al*^2016^), *Brazil, *Chile, *Ecuador
